# Conditional cash transfers and the reduction in partner violence for young women: an investigation of causal pathways using evidence from a randomized experiment in South Africa (HPTN 068)

**DOI:** 10.1002/jia2.25043

**Published:** 2018-02-27

**Authors:** Kelly N Kilburn, Audrey Pettifor, Jessie K Edwards, Amanda Selin, Rhian Twine, Catherine MacPhail, Ryan Wagner, James P Hughes, Jing Wang, Kathleen Kahn

**Affiliations:** ^1^ University of North Carolina Chapel Hill United States; ^2^ MRC/Wits Rural Public Health and Health Transitions Research Unit (Agincourt) University of the Witwatersrand Johannesburg South Africa; ^3^ University of Wollongong Wollongong NSW Australia; ^4^ Wits Reproductive Health and HIV Institute University of the Witwatersrand Johannesburg South Africa; ^5^ Umeå Centre for Global Health Research Umeå University Umeå Sweden; ^6^ Statistical Center for HIV/AIDS Research and Prevention (SCHARP) Seattle United States; ^7^ University of Washington Seattle Washington; ^8^ INDEPTH Network Accra Ghana

**Keywords:** intimate partner violence, cash transfers, gender, South Africa, HIV prevention

## Abstract

**Introduction:**

Evidence has shown that the experience of violence by a partner has important influences on women's risk of HIV acquisition. Using a randomized experiment in northeast South Africa, we found that a conditional cash transfer (CCT) targeted to poor girls in high school reduced the risk of physical intimate partner violence (IPV) in the past 12 months by 34%. The purpose of this analysis is to understand the pathways through which the CCT affects IPV.

**Methods:**

HPTN 068 was a phase 3, randomized controlled trial in rural Mpumalanga province, South Africa. Eligible young women (aged 13–20) and their parents or guardians were randomly assigned (1:1) to either receive a monthly cash transfer conditional on monthly high school attendance or no cash transfer. Between 2011 and 2015, participants (N = 2,448) were interviewed at baseline, then at annual follow‐up visits at 12, 24 and 36 months. The total effect of the CCT on IPV was estimated using a GEE log‐binomial regression model. We then estimated controlled direct effects to examine mediation of direct effects through intermediate pathways. Mediators include sexual partnership measures, the sexual relationship power scale, and household consumption measures.

**Results:**

We found evidence that the CCT works in part through delaying sexual debut or reducing the number of sexual partners. The intervention interacts with these mediators leading to larger reductions in IPV risk compared to the total effect of the CCT on any physical IPV [RR 0.66, CI(95%):0.59–0.74]. The largest reductions are seen when we estimate the controlled direct effect under no sexual debut [RR 0.57, CI(95%):0.48–0.65] or under no sexual partner in the last 12 months [RR 0.53, CI(95%):0.46–0.60].

**Conclusions:**

Results indicate that a CCT for high school girls has protective effects on their experience of IPV and that the effect is due in part to girls choosing not to engage in sexual partnerships, thereby reducing the opportunity for IPV. As a lower exposure to IPV and safer sexual behaviours also protect against HIV acquisition, this study adds to the growing body of evidence on how cash transfers may reduce young women's HIV risk.

## 1 Introduction

Violence against women, and specifically intimate partner violence (IPV), is a major global public health problem, causing significant morbidity and mortality worldwide 1. Around a third of women globally have experienced IPV 2 and South Africa, the location of this study, is no exception 3, 4. Partner violence in South Africa is particularly a problem for young women, putting them at risk for sexual and reproductive health issues including HIV infection 5. IPV can be a direct cause of HIV transmission through forced or coercive sex with a HIV‐positive male but can also indirectly lead to HIV transmission by limiting young women's ability to negotiate and practice safe sexual behaviours such as using condoms 5, 6, 7. Moreover, poverty can exacerbate young women's risk for both IPV and HIV as it often heightens this gendered power imbalance by pressuring young women to engage in transactional sex 7, 8, 9. Given the critical intersections of HIV and IPV, the success of HIV prevention interventions may be conditional upon changes in gendered power inequalities.

The importance of addressing these interconnections—those among health, gender inequalities and poverty—has even been articulated in the United Nations Sustainable Development Goals (SDGs) in order to advance the global development agenda. In context of HIV prevention for female adolescents in South Africa, a focus on combination interventions which work towards reducing national poverty (SDG 1.2) and gender inequality (SDG 5), particularly intimate partner violence (SDG 5.2.1), may be the most imperative to reduce new HIV infections (SDG 3.3.1). Social protection programmes have become an explicit part of the development agenda (SDG 1.3) to reduce national poverty and lately, these programmes—particularly cash transfers—have also been promoted for HIV prevention. The theory is that cash transfers may reduce women's HIV risk as they can address poverty as structural driver of risk and because cash transfers may be an effective vehicle for empowering women and lead to safer sexual behaviours 10, 11, 12, 13. Moreover, cash transfers often increase children's educational attendance (SDG 4.1) with even stronger effects when the money is tied to an explicit condition 13. As education can also be empowering for girls, providing the cash conditional on school attendance may be an important mechanism for reducing young women's vulnerability to HIV and IPV 14, 15, 16.

In this analysis, we examine how a conditional cash transfer (CCT), HIV Prevention Trial Network (HPTN) 068 study, helped address these intersecting issues in poor, rural South Africa. HPTN 068 was an experimental intervention for HIV prevention that provided monthly cash transfers to young women and their households conditional high school attendance by the young woman. The main findings of HPTN 068 (published in the Lancet) revealed that the CCT had no significant effect on HIV incidence but did reduce young women's risk of IPV by 34 percent 17. While similar evidence has been found in other studies of cash transfers 18, 19, 20, 21, most evidence comes from Latin America and focuses on older women. Moreover, little is known about how these programmes work to prevent IPV 21. In this paper, we build upon the Lancet findings to investigate the causal pathways through which a CCT intervention targeted to young women in South Africa works to reduce IPV; concentrating on perceived sexual relationship power, sexual behaviours and economic wellbeing.

## 2 Methods

### 2.1 Study site and design

The HPTN 068 study site is in the Mpumalanga province in northeast South Africa. Villages in our study are located within the Agincourt Health and Socio‐Demographic Surveillance Systems (AHDSS) catchment area—a rural but densely populated and characterized by high poverty and high HIV prevalence 22, 23. A 2010 population‐based HIV prevalence survey undertaken in Agincourt found HIV prevalence rises significantly among young women of similar age to our study sample—from 5.5% among 15–19 year olds to 27% among 20–24 year olds 23. Incidence among young women from the HPTN 068 study was <2% (per person‐year) 17, which was low considering a 4.5% incidence rate among black African females aged 20–34 from a recent national survey in South Africa 24.

The study was designed as a phase III randomized control trial to test the effectiveness of CCTs for HIV prevention among young women (aged 13–20) attending high school. Enrolled participants were randomly assigned to the treatment arm, and participants and their parents or guardians received monthly cash transfers of 100 and 200 Rand (R) respectively (or roughly US$ 10 and US$ 20 using 2012 the conversion rates). At baseline, monthly per capita household expenditure was R295 so transfer amounts made up a significant proportion of pre‐programme consumption. For both the young woman and the parent or guardian, transfer funds were deposited directly into respective bank accounts. Cash transfers were conditional on the young woman attending at least 80% of school days during the month. As long as the young woman was eligible to be in school and met the attendance criteria, she could receive the transfer for up to 3 years.

### 2.2 Eligibility and randomization

To be eligible for the study, young women had to be aged 13–20 years and enrolled in a participating high school (grades 8, 9, 10, or 11) in the study location. They also had to be unmarried, not pregnant, able to read, living with at least one parent or guardian, willing to take an HIV and herpes simplex virus (HSV)‐2 test, and have or be able to open a bank account (or post office account). Between March 2011 and December 2012, a total of 10,134 young women were screened from the ADHSS population, and 2,537 were found eligible and enrolled 25. After young women were recruited to the study, participants completed an Audio Computer‐Assisted Self‐Interview (ACASI) and HIV and HSV‐2 testing, which included pre and post‐test HIV counselling. After baseline assessments, young women (and their parent or guardian) were individually randomized (1:1) to either the treatment group (monthly cash transfer conditional on school attendance) or control group (no cash). All participants were then reassessed annually at 12, 24, and 36 months until they graduated from high school or the study ended, whichever came first. Each visit included the ACASI, HIV and HSV‐2 testing (if negative at the previous visit), and pre and post‐test HIV counselling. A household survey was also completed with parents or guardians at baseline and each follow‐up visit. Consent for study participation was obtained at the home visits with written informed consent from both young women and her parent or guardian. Written assent was obtained for female participants under 18 years old. Institutional Review Board approval for this study was obtained from the University of North Carolina at Chapel Hill and the University of the Witwatersrand Human Research Ethics Committee as well as the Provincial Department of Health's Research Ethics Committee.

### 2.3 Measures

The ACASI asked respondents about sexual and physical IPV experiences regardless of whether they reported having a sexual partner. “Forced sex” is an indicator any experience of forced sex by a partner in the previous 12 months. “Any physical IPV” is an indicator for any experience of physical intimate partner violence in the previous 12 months as defined by the WHO 2;

Has a partner (responses are yes or no):

1. Slapped you or thrown something at you that could hurt you?
2. Pushed you or shoved you?
3. Hit you with a fist or with something else that could hurt?
4. Kicked you, dragged you, or beaten you up?
5. Choked or burnt you on purpose?
6. Threatened to use or actually used a gun, knife, or other weapon against you?

We also created indicators for moderate and severe IPV as defined by the WHO, where moderate IPV indicates any experience of violence from items 1 or 2 and severe IPV indicates any experience of violence from items 3–6. Variables are not mutually exclusive; young women could experience both moderate and severe IPV.

Mediation pathways we examined included sexual behaviours, perceived sexual relationship power and household economic wellbeing. We chose to explore these variables based on hypothesized conceptual pathways through which the cash transfer might reduce IPV 21, 26. For one, sexual behaviours that increase the risk of IPV (e.g. transactional sex) may be driven in part by poverty, including relative poverty. Individual transfers to young women may reduce these behaviours if the money improves outcomes such as food security or consumption for “symbolic purposes” 27. In addition, young women are more susceptible to violence if they are economically dependent on their male partners. But, with their own source of money, relationship power dynamics may change or women may feel more empowered to leave abusive relationships. Finally, income transfers at the household level have been shown to reduce economic insecurity, which in turn may further lower young women's risk of IPV by changing household time‐use decisions. For young women, this may include more time spent in school and at home vs. high‐risk settings such as unsafe work environments.

Sexual behaviour measures included an indicator for sexual debut (vaginal or anal) after baseline, an indicator for whether the girl had any sexual partners in the past 12 months, and the number of sexual partners in the last 12 months. Sexual relationship power was defined only for young women that reported ever having had sex and operationalized with the Sexual Relationship Power Scale (SRPS), which measures constructs of relationship control and decision‐making dominance 28, 29. We looked at the continuous scale (higher scores indicate greater perceived empowerment) and similar to other studies that use the SRPS, we split the scale into terciles based on scores from all waves. Economic measures included per capita household expenditure (in logarithms) and an indicator for being in the top quartile of per capita household expenditure.

### 2.4 Analysis methods

We began with an intention‐to‐treat (ITT) analysis of the total effect of the CCT on IPV measures and on potential mediators for all participants with an HIV‐negative status at baseline. Intervention effects were modelled using generalized estimating equation (GEE) models with robust variance to account for repeated observations on study participants. We estimated risk ratios using log‐binomial regressions for binary (and count) mediators. For continuous mediators (SRPS and log per capita expenditure), coefficients are calculated using a Gaussian distribution. All models control for participant's age at baseline. In addition, sexual debut was modelled using a discrete time survival analysis and includes dummy variables for study visits.

To explore mediation of the effect of the CCT on IPV, we used the counterfactual approach to causal mediation 30, 31, 32 where we estimate the risk of IPV, Y, for everyone under each possible exposure; treatment, Y(1), and control, Y(0). Only mediators that were significantly impacted by treatment were considered for mediation analysis. We estimated the controlled direct effect (CDE) of the CCT, which expresses the effect of keeping the mediator controlled at level M for everyone but switching exposure from control, Y(0), to treatment, Y(1).

CDE(M)=E[Y(1,M)]/E[Y(0,M)]

In general, CDEs are used to estimate what the difference in the effect of the exposure would be if you could impose a mediator intervention. In our study, CDEs represent the hypothetical risk reduction if we were able to set our mediators at a more protective level (e.g. reducing sexual partners).

We estimated the CDE using the parametric g‐formula 32. In the first step, we fit log‐binomial models for the effect of CCT on IPV at each time point, including terms for each mediator, treatment‐mediator interactions, and baseline levels of confounders (see Appendix S1 for details). We then used the coefficients from this model to estimate the predicted probabilities of the outcome under each hypothetical intervention on the exposure (treatment or control) and mediator. We report risk ratios for each contrast of interest as the ratio of the average predicted outcome probability under each hypothetical intervention compared. Standard errors of the risk ratios were estimated as the standard deviation of the point estimate from 5,000 bootstrap samples of the observed data 34.

## 3 Results

### 3.1 Baseline data

Baseline descriptive statistics for study participants (total and by study arm) are provided in Table 1. We exclude baseline HIV positive or unknown cases (N = 85) from our analysis leaving a baseline sample of 2,448 HIV‐negative young women. All young women participating in the study were South African and of black race/ethnicity. Young women had a median age of 15 years (IQR 14–17) and were distributed equally across all school grades (8–11). All demographic and outcome variables in Table 1 were tested for baseline balance and we found no significant differences in means between study arms at the 10 percent significance level. In addition, the study arms were also balanced on other key behavioural outcomes, including the main outcomes of HIV and HSV‐2 infection status 17.

**Table 1:** Baseline demographics and outcomes for young women study participants by treatment arm

	Total (n = 2,448)	Treatment group (n = 1,225)	Control group (n = 1,223)
	N (%) or Median (IQR)		
Age (years)	15 (14 to 17)	15 (14 to 17)	15 (14 to 16)
School grade enrolment			
Grade 8	614 (25)	310 (25)	304 (25)
Grade 9	669 (27)	321 (26)	348 (28)
Grade 10	677 (28)	347 (28)	330 (27)
Grade 11	488 (20)	247 (20)	241 (20)
Ever physical IPV	415 (17)	219 (18)	196 (16)
Ever forced sex	73 (3.0)	33 (2.7)	40 (3.3)
Any physical IPV in past 12 months	254 (11)	136 (11)	118 (10)
Ever vaginal or anal sex	649 (27)	324 (26)	325 (27)
Any sexual partner in past 12 months	645 (27)	316 (26)	329 (27)
Number of sexual partners in past 12 months			
0 partners	1,773 (73)	893 (74)	880 (73)
1 partner	504 (21)	247 (20)	257 (21)
≥2 partners	141 (6)	69 (6)	72 (6)
Sexual relationship power scale (SRPS)^a^	28 (23 to 32)	28 (23 to 32)	29 (24 to 32)
Highest tercile SRPS (34–36)	132/653 (20)	70/324 (22)	62/329 (19)
Log PC Expenditure (2.9–9.7)	5.7 (5.2 to 6.2)	5.7 (5.2 to 6.2)	5.7 (5.2 to 6.2)

No significant differences (*p* < 0.1) found between treatment and control outcomes at baseline.

a SRPS is only reported for girls who had been sexually active (n = 697), scale range is from 1 to 36 with higher scores representing greater empowerment.

At baseline, 17 percent of all young women in the study reported ever having experienced physical IPV by a partner and 11 percent had experienced some form of physical IPV in the past 12 months. The majority of study participants were not sexually active at baseline, only 27 percent reported ever having had sex (vaginal or anal). In addition, only around 3 percent had ever experienced forced sex.

Table 2 shows ITT programme impacts on all IPV outcomes including the main effects of whether a participant experienced forced sex by a partner (row 1) and physical IPV (row 2) in the past 12 months. We found no effect on forced sex, but, as reported in the Lancet 17, the programme resulted in a significant reduction in physical IPV. Young women in the treatment group have a 34 percent lower risk of IPV (RR 0.66), significant at the 99.9% CI level.

**Table 2:** Intent to Treat Impacts of the CCT on IPV among young women enrolled in HPTN 068

	Treatment	Obs.^a^	Control	Obs.^a^	Risk ratio (95% CI)
Forced sex	58 (2.5%)	2,282	46 (2.2%)	2,062	1.13 (0.75–1.70)
Any physical IPV	473 (18.5%)	2,559	636 (27.8%)	2,290	0.66*** (0.59–0.74)
Individual items					
Slapped or threw something	377 (14.7%)	2,564	519 (22.6%)	2,295	0.65*** (0.57–0.74)
Pushed or shoved	282 (11.0%)	2,564	391 (17.1%)	2,292	0.64*** (0.56–0.74)
Hit with fist/another item	203 (7.9%)	2,563	309 (13.5%)	2,294	0.59*** (0.50–0.69)
Kicked, dragged, or beaten up	198 (7.7%)	2,562	272 (11.9%)	2,293	0.65*** (0.54–0.78)
Choked or burnt	148 (5.8%)	2,561	222 (9.7%)	2,293	0.60*** (0.48–0.73)
Threatened or used gun/another weapon	140 (5.5%)	2,563	205 (8.9%)	2,293	0.61*** (0.49–0.75)
Severity					
Moderate	445 (17.4%)	2,559	591 (25.8%)	2,290	0.67*** (0.60–0.75)
Severe	260 (10.2%)	2,559	367 (16.0%)	2,290	0.63*** (0.54–0.74)

Data are n (%) unless otherwise stated. RRs from GEE log‐binomial model adjusted for age.

^+^ *p *< 0.10, *p < 0.05, **p < 0.01, ***p < 0.001.

a Observations over the three follow‐up study visits, out of total N of 2,302 study participants (1,209 treatment and 1,093 control) followed up for at least one visit after baseline.

Below the main treatment effects, we break down any physical IPV into its component parts and find that treatment effect is robust across each of these different specifications. For the six separate acts of physical violence, risk ratios for each measure are similar to the overall impact (ranging from 0.59–0.65) and significant at the 99.9% CI level. In addition, results are just as robust to categorization of IPV into moderate and severe categories (*p*‐values < 0.001).

Earlier we described the potential pathways we examined related to sexual partnerships, relationship power and economic wellbeing to explain the effect of the CCT on reducing risk of IPV. Table 3 shows the treatment impacts on these pathways. As a necessary condition for mediation, we should find that the CCT directly impacts these pathways.

**Table 3:** Intent to Treat Impacts of the CCT on Hypothesized Mediators among young women enrolled in HPTN 068

	Treatment	Obs.^a^	Control	Obs.^a^	RR, IRR, or Coef. (95% CI)
Sexual debut^b^^,^ c	245 (13.9%)	1,762	250 (16.6%)	1,510	RR 0.83* (0.71–0.98)
Number of sexual partners in the last 12 months (Mean, SD)	0.41 (0.7)	2,574	0.46 (0.8)	2,292	IRR 0.86** (0.78–0.96)
Any sexual partner in last 12 months	841 (33%)	2,574	824 (36%)	2,292	RR 0.90* (0.83–0.99)
Over one sexual partner in last 12 months	147 (5.7%)	2,574	149 (6.3%)	2,292	RR 0.88 (0.68–1.09)
SRPS^d^^,^ e (Mean, SD)	28.6 (7.0)	936	28.6 (6.7)	916	Coef. 0.06 (−0.63–0.75)
High SRPS^e^^,^ f	296 (31.6%)	936	264 (28.8%)	916	RR 1.12 (0.96–1.30)
Log per capita expenditure (Mean, SD)	6.0 (0.7)	2,583	6.0 (0.7)	2,290	Coef. 0.03 (−0.02–0.08)
Top quartile per capita expenditure	670 (26.9%)	2,583	552 (24.1%)	2,290	RR 1.09 (0.97–1.22)

Data are n (%) unless otherwise stated. Estimates from GEE models adjusted for age (sexual debut also adjusts for time dummies).

RR, risk ratio; IRR, incident rate ratio; Coef, coefficient.

^+^ *p* < 0.10, *p < 0.05, **p < 0.01, ***p < 0.001.

a Observations over the three follow‐up study visits, out of total N of 2,302 study participants (1,209 treatment and 1,093 control) followed up for at least one visit after baseline.

b Among those who had not debuted before baseline.

c Per person‐visit.

d Sexual Relationship Power Scale, scores ranges from 1 to 36.

e Only girls that reported having sex either at some point at that visit or before, or if she reported having a partner after being asked IPV questions.

f High SRPS is top tercile (≥34) and compared to low or moderate SRPS score.

Using a discrete time‐survival analysis, we find that there is a reduced risk of sexual debut (RR 0.83), significant at the 95% CI level. In other words, the CCT has a cumulative, protective effect of first sex at each study visit. It is important to note that in the main trial findings, no significant difference was found in sexual debut between study arms using a cox proportional hazards model to measure risk of debut over the entire study period 17. We chose to examine the cumulative risk of debut for this analysis because we are interested in the pathways that affect incident IPV by visit. Another difference is that we excluded cases of debut if women reported an age of first sex that was younger than their age at baseline. However, sensitivity analysis confirms that results are robust to model choice and not dependent on the inclusion or exclusion of these cases.

In addition, the programme had a positive effect of reducing sexual partnerships during the study. Since partner number is a count variable and most sexually active girls had only one partner we estimated the incident rate ratio and find that being in the intervention resulted in a significantly lower risk of having an additional partner (IRR 0.86). Similarly, participants in the treatment arm had a 9% lower risk of having any sexual partner in the last 12 months (*p*‐value < 0.05).

In addition, we examined the effect of CCT on young women’ perceived relationship power using the SRPS. As only young women that reported having sexual partnerships have SRPS scores, the sample is reduced considerably (Table 3). We found no significant effect of the CCT intervention on either the continuous SRPS scale or the likelihood of scoring in the top tercile (a score of 34 or greater). Lastly, we found no effect of the CCT on logged per capita household expenditure or on the likelihood of being in the top quartile of per capita expenditure.

To examine causal mediation of the CCT on IPV, we used the three partnership measures that were significantly affected by treatment (sexual debut, any sexual partner and number of sexual partners). Since treatment effects in Table 2 are robust to different specifications of IPV, we used any IPV as our key outcome to analyse mediation. We estimated CDEs for each mediator‐outcome pair using the G‐computation formula for mediation (Table 4). For each mediator intervention defined in the left column, we show the CDE broken into the absolute risk of IPV (for each arm) and the risk ratio.

**Table 4:** Controlled direct effects of mediator interventions on physical intimate partner violence using parametric G‐computation formula

Mediator intervention	Risk (%) (T = treatment C = control)	Risk ratio (95% CI)	No mediator intervention
No sexual debut	T = 14.8	0.57 (0.48–0.65)	Risk (%) T = 18.5 C = 27.8 (RR 0.66)
	C = 26.2		
No sexual partner (past 12 months)	T = 14.8	0.53 (0.46–0.60)	
	C = 27.9		
Reduce sexual partners by one (past 12 months)	T = 16.2	0.59 (0.53–0.66)	
	C = 27.4		

Adjusted for age and baseline level of mediator. Binary outcomes estimated using a GEE log‐linear model and robust variance. Number of sexual partners estimated using a linear regression model with standard errors clustered at the individual level. Effects estimated from Monte Carlo sample (N = 5,000) with 95% CIs calculated from standard deviations of point estimates.

Compared to total effect of RR 0.66 (shown in the last column), we find that each mediator intervention additionally lowered the risk of IPV for young women in treatment. The first mediator intervention sets all young women to no sexual debut after baseline resulting in a risk ratio of 0.57, approximately 9 percentage points lower than the total effect. The second row shows the effect of setting all young women in the study to having no sexual partner. This intervention results in the smallest risk ratio (0.53), which is more than 10 percentage points lower than the original total effect. In the last row, we tested a less restrictive mediation scenario by reducing sexual partnerships by 1 (for all those with 1 or more partners). This also reduced the risk of IPV by 7 percentage points (RR 0.59) from total effect. Moreover, for each mediator intervention, we see that the absolute risk of IPV is lower for young women in the treatment group compared to the original risk in the right‐hand column (18.5 percent). For young women in the control group, however, the risk is about the same as the original (27.8 percent). Therefore, the interaction of the mediator and treatment is driving the result—intervening on the mediator works to reduce risk of IPV only in combination with the CCT.

## 4 Discussion

This study explored how a cash transfer intervention given to young women in South Africa conditional on school attendance reduced the risk of intimate partner violence. We first showed treatment effects were significant for all types of physical violence, but that there was no coinciding effect on sexual violence. The CCT also had significant impact on sexual partnerships including sexual debut by visit, any partner in the last 12 months, and number of partners in the last 12 months. We then used the G‐formula for mediation analysis to estimate CDEs for each of these sexual partnership mediators. Our findings indicate that while the intervention had a strong direct effect on physical IPV, it also had a significant interactive effect with sexual partnership behaviours, leading to even lower risks of IPV. While CDE estimates are hypothetical effects, they provide insight into how mediators work and they have the potential to inform policy about how complementary interventions might work using observed effects 35.

This evidence adds to the growing body of literature showing that CCTs can reduce the risk of IPV 18, 19, 20, 21. However, whereas the current evidence focuses on older women who are married or cohabiting with their intimate partner, our study provides new evidence on the effect for adolescent girls and young women still living at home and attending school. Developmentally, this may be important for adolescents’ successful transition into adulthood since for most of these young women this is their first relationship, thus setting patterns for experiencing IPV in future relationships. In addition, our results are important because there was concern that the cash could lead to more conflict within relationships and increase risk of IPV 18, 21. Instead, we provide evidence for the supporting role that economic interventions can have on young women's risk of IPV and potential effect on HIV transmission given the critical intersections of the two.

Despite our finding that the CCT reduced risky sexual behaviours and the link between IPV and HIV in South Africa 5, the CCT intervention did not lead to a parallel reduction in new HIV infections 17. A likely reason is that the intervention did not lead to differential rates of school attendance between treatment and control groups—it was very high for both groups—and that schooling itself has a protective effect on HIV. Indeed, the study found school attendance itself had a significant effect on HIV incidence 17. It is also possible that the reductions in sexual debut and partner number were not enough to affect HIV incidence (or to enable discernible differences in incidence) given fairly low rates in both arms during the trial. This is also true for forced sex—sexual violence was much less prevalent compared to physical violence at baseline, which could help explain the null findings.

Besides the inability to link impacts on IPV to HIV incidence, our analysis of mediation pathways could be improved with better measures. For one, the SRPS is only available for women who reported ever having sex. Unlike evidence from other studies linking reductions in IPV to relationship power dynamic changes 20, the CCT does not have an impact on sexual relationship power within current relationships. This may again speak to the point that young women are choosing to not engage in any sexual relationships (which itself could be the ultimate form of sexual relationship power). However, since most young women were still in school and living at home as opposed to cohabiting with partners, potential impacts in an older, married or largely cohabiting population may be different. In addition, despite the fact there was no treatment effect on household expenditures, economic wellbeing likely plays a role since the direct effect on IPV is so robust. Qualitative evidence indicates that the cash had positive effects on young women's feelings of independence and financial empowerment 36. Therefore, a more universal measure of empowerment could improve our understanding of how the intervention affects young women’ attitudes and decision‐making.

Despite these limitations, there are noteworthy strengths of the study including the randomized study design and longitudinal data that not only allows for causal estimation of treatment effects but provides stronger causal assumptions for estimation of mediation effects. In addition, we believe our findings have broader generalizability across poor, rural settings in South Africa given the high coverage of other social protection programmes.

## 5 Conclusions

This study is the first to investigate the causal pathways through which a cash transfer programme reduced IPV among young women. In contrast to other studies with older women, we do not find that the cash works through changing power dynamics within existing partnerships but rather the effect was boosted through young women choosing not to engage in sexual partnerships. Furthermore, there is a remaining direct effect of the CCT on physical IPV. In terms of the global development agenda, these results show improved SDG outcomes for gender equality, specifically the reduction in physical violence against women (SDG 5.2.1). But, since there was no effect on HIV incidence (SDG 3.3.1) or sexual violence (also SDG 5.2.1), is not clear that CCTs alone are an effective strategy for HIV prevention for young women in South Africa.

Nonetheless, given the critical intersections of HIV and IPV in high‐poverty contexts*,* this study adds to the growing evidence on the role of social protection in reducing poor young women's risk of HIV 12, 13, 37. In the context of South Africa, social protection already reaches many vulnerable groups, including poor adolescents under 18 covered under the Child Support Grant (CSG). Recent evidence has shown that the CSG is similarly protective of HIV risk behaviours in adolescents 18 and under 37, 38. In view of our findings, continued cash payments for young women older than 18 could extend this protective effect, especially considering youth between 18 and 24 in South Africa are regularly still in school or unemployed and this is the age when HIV incidence starts to take off 23. Extending or expanding existing programmes is clearly not an insignificant policy decision, but greater inclusion of vulnerable young people into social protection schemes could have important implications for HIV prevention and the health of the next generation.

Although our findings come from an experimental intervention, cash transfers play a major role in many SSA governments’ social protection schemes 39. Consequently, these findings illustrate the potential for current social protection programmes in the SSA region to achieve important progress in SDGs, which builds on other recent findings demonstrating how social protection in South Africa is benefiting adolescent across many SDGs 40. In particular, our results highlight the benefits of combining age and gender‐sensitive targeting with structural interventions to achieve the SDG agenda as it relates to adolescent HIV prevention. In this regard, policy‐makers across social development and public health ministries would do well to work together to integrate targeted public health interventions for young women into social protection programmes in order to harness the synergistic power of the two and improve SDGs related to poverty, health and gender equality.

## Competing interests

The authors have no competing interests to declare.

## Authors’ contributions

KNK and JKE contributed to data analysis, data interpretation and writing of this manuscript. AP and AS contributed to study design, development of data collection instruments and the protocol, study oversight and implementation, data interpretation, data analysis and writing of this manuscript.CM and KK contributed to study design, development of data collection instruments and the protocol, study oversight and implementation, and editing of this manuscript. RT contributed to the protocol development, study implementation and oversight, and editing of the manuscript. JPH contributed to study design, development of the protocol, data interpretation and editing of this manuscript. JW contributed to data analysis, data interpretation and editing of the manuscript. RW contributed to study implementation and oversight, and editing of the manuscript.

## Supporting information


**Appendix S1** Models used to estimate controlled direct effects.
