# Supplementary material for: Conditional cash transfers and the reduction in partner violence for young women: an investigation of causal pathways using evidence from a randomized experiment in South Africa (HPTN 068)
Source: J Int AIDS Soc. 2018 Feb 27;21(Suppl Suppl 1):e25043. doi: 10.1002/jia2.25043 (PMC5978692; doi:10.1002/jia2.25043)
Supplement: Supplementary file 1 — Appendix S1 Models used to estimate controlled direct effects. [file JIA2-21-e25043-s001.docx]

**Online Appendix**

**Models used to Estimate Controlled Direct Effects**

We first estimate model (1) and (2) (below) for each mediator where A represents the treatment, Y the outcome, M the mediator, and C additional covariates. Model (2) is the same as model (1) but also includes treatment-mediator interactions (AM). These models were proposed by Valeri and Vanderweele (2013) and are defined for binary outcomes and mediators. Since outcomes are relatively common we use log-linear models to estimate risk ratios [30]. Using the counterfactual framework, we then estimate controlled direct effects (CDE) from the regression parameters in either Models (1) or (2) depending upon whether there is an interaction effect (AM).

1. $log\{E\left( Y | A,M,C \right)\}=\theta_{0}+\theta_{1}A+\theta_{2}M+\theta_{3}C$
2. $log\{E\left( Y | A,M,C \right)\}=\theta_{0}+\theta_{1}A+\theta_{2}M+\theta_{3}AM+\theta_{4}C$

**Table A1. Causal Mediation Models with and without Interaction Effects**

|  | Sexual debut | | Any sexual partner  in past 12 months | | Number of Sexual Partners | |
| --- | --- | --- | --- | --- | --- | --- |
|  | (1) | (2) | (3) | (4) | (5) | (6) |
| Treatment | 0.68*** | 0.56*** | 0.68*** | 0.54*** | 0.68*** | 0.59*** |
|  | (0.61 - 0.76) | (0.47 - 0.66) | (0.61 - 0.76) | (0.46 - 0.63) | (0.61 - 0.76) | (0.51 - 0.67) |
| Mediator | 1.46*** | 1.27** | 1.36*** | 1.13 | 1.20*** | 1.10* |
|  | (1.30 - 1.64) | (1.10 - 1.47) | (1.20 - 1.53) | (0.97 - 1.31) | (1.13 - 1.28) | (1.01 - 1.20) |
| Interaction |  | 1.41** |  | 1.58*** |  | 1.236** |
|  |  | (1.13 - 1.76) |  | (1.27 – 1.96) |  | (1.11 - 1.37) |
| Observations | 4,848 | 4,848 | 4,806 | 4,806 | 4,806 | 4,806 |

Notes: Adjusted for age and baseline mediator value; coefficients are RR (95%CI) estimated using GEE models with log-linear specification and robust SEs. Mediators labeled at the top are included into the regressions in columns (1), (3), and (5) while mediator and mediator-treatment interactions are also included in columns (2), (4), and (6)

^+^ pvalue<0.10, *pvalue<0.05, **pvalue<0.01, ***pvalue<0.001

Results in Table A1 show that using model (1) in columns 1,3, and 5, treatment effects in the first row remain similar in size and significance to the total treatment effect of RR 0.66 (CI: 0.59 - 0.74). Additionally, all mediator coefficients are significant and show that any engagement in a sexual partnership increases the risk of physical IPV. Using model (2) in columns 2, 4, and 6, we also see significant effects on each mediator-treatment interaction terms. Therefore, we used model (2) to estimate CDEs in the paper.

**Identifiability of Casual Mediation Effects**

In order to interpret causal mediation effects under the counterfactual approach, certain identifiability assumptions are required: no unmeasured confounding between 1) treatment and the outcome, 2) treatment and the mediator, 3) mediator and the outcome (including no confounder affected by treatment) [30]. Randomization to treatment arms in this study assures assumptions (1) and (2) are met, but it does not guarantee that no confounding holds for the mediator-outcome relationship in assumption (3) because the mediator was not randomized. Therefore, to account for potential confounding issues between the mediator-outcome relationship, we control for observed pretreatment covariates including baseline mediator levels and participant age in all models [31].
